# Serum uric acid and triglycerides in clear cell renal cell carcinoma: A restrospective cross-sectional study

**DOI:** 10.1097/MD.0000000000034645

**Published:** 2023-09-15

**Authors:** Dong Yue, Jun Tian

**Affiliations:** a Department of Organ Transplantation, Qilu Hospital, Cheeloo College of Medicine, Shandong University, Lixia District, Jinan, Shandong Province, China; b Department of Urology, Affiliated Hospital of Jining Medical University, Jining, Shandong Province, China.

**Keywords:** clear cell renal cell carcinoma, lipids, serum uric acid, triglycerides

## Abstract

The available evidence on the relationship between serum uric acid and triglycerides in clear cell renal cell carcinoma (ccRCC) is limited. The objective of this study is to investigate whether there is an independent association between serum uric acid and triglycerides in ccRCC, while adjusting for other variables. This cross-sectional study involved 1018 participants with ccRCC, who were admitted to a hospital in China from December 1st, 2013 to January 1st, 2023. The main independent variable investigated was serum uric acid, which was measured at baseline. The dependent variable was triglycerides. Covariates considered in this study included age, sex, body mass index, smoking status, hypertension, diabetes, coronary disease, High-density lipoprotein cholesterol, Low-density lipoprotein cholesterol, Total cholesterol, Blood urea nitrogen, and Creatinine. The study included 1018 participants with an average age of 56.92 ± 10.88 years old, and approximately 68% of them were male. The fully-adjusted linear regression analysis indicated a positive association between serum uric acid levels (100μmol/L) and triglyceride levels (mmol/L) after adjusting for confounding factors (β = 0.13, 95% CI [0.07, 0.18]). Furthermore, a smooth curve was constructed between serum uric acid and triglycerides based on the fully-adjusted model. In patients with ccRCC, there was a positive association between serum uric acid and triglycerides.

## 1. Introduction

Renal cell carcinoma (RCC) is the 6th most commonly diagnosed cancer in men and the 10th most commonly diagnosed cancer in women, accounting for approximately 5% and 3% of all cancer diagnoses, respectively.^[1]^ According to the American Cancer Society, an estimated 81,800 new cases of RCC will be diagnosed by the end of 2023, and more than 14,890 deaths will be attributed to RCC in the USA alone.^[2]^ The incidence of RCC varies among different geographic regions, countries, and regions, with a higher incidence in developed countries compared to developing countries.^[3]^ Common risk factors for RCC include smoking, obesity, hypertension, long-term use of certain medications (such as analgesics), and genetic factors.^[4]^ The most common subtype of RCC is clear cell RCC (ccRCC), which accounts for approximately 75% of all cases.^[5]^ It is an aggressive cancer that originates from the proximal convoluted tubule. After initial treatment of a localized tumor, there is often a recurrence rate of up to 40%.^[6]^ When it metastasizes, ccRCC is associated with a high mortality rate.^[7]^

So far, the potential mechanism of renal cell carcinoma remains to be fully elucidated, in a metabolomic analysis of ccRCC, it was found that metabolites involved in glycolysis pathway were present in over 2-fold higher concentrations compared to normal kidney tissues. GSH metabolism-related metabolites, such as cysteine, γ-glutamyl cysteine, and GSH, also showed increased levels in the late-stage ccRCC cases. These metabolites have been found to be associated with worse survival outcomes in ccRCC patients.^[8]^ Recent findings supported that the importance of synthesis and metabolism of lipids in the carcinogenesis and biology of ccRCC.^[9]^ However, the evidence regarding the relationship between metabolic abnormalities and renal cell carcinoma is limited.

Serum uric acid (SUA) is the end product of purine metabolism.^[10]^ While elevated levels of SUA have been suggested to be associated with an increased risk of cancer as a marker of chronic inflammation, it is also known for its antioxidant properties which could potentially have an anticancer effect.^[11]^ Similar results were obtained for postmenopausal breast cancer, hepatobiliary-pancreatic cancer, colorectal cancer, and kidney cancer.^[12–15]^ However, Some studies have found no evidence to support the theory that serum urate is a modifiable risk factor for respiratory health or lung cancer.^[16]^ Triglycerides (TGs) are major component of body fat and serve as an energy reservoir. They are composed of an ester derived from glycerol and 3 free fatty acids.^[17]^ Targeting triglyceride metabolism were reported in breast cancer, lung cancer, prostate cancer.^[18–20]^some studies suggested SUA obtained at baseline was related to TGs.^[21,22]^ In contrast, In some other studies, no association between SUA and TGs were found in multivariable analyses.^[23]^

Since previous studies on the relationship between SUA and TGs have varied in terms of research design, target population, and data analysis, additional studies are still needed to better understand this relationship. Therefore, this study aims to investigate whether there is an independent association between SUA and TGs in ccRCC.

## 2. Participants and methods

### 2.1. Study design

This cross-sectional study aims to explore the correlation between SUA and TGs. The study use baseline SUA levels as the independent variable and TGs as the dependent variable.

### 2.2. Study population

Participants with newly-diagnosed ccRCC were consecutively and non-selectively enrolled from the Department of Urology at the Affiliated Hospital of Jining Medical University, located in Jining city, China. No identifiable participant data was included in the data collection process to protect patient privacy, and the data was obtained through the hospital’s electronic medical record system. As this study was retrospective in nature, participant informed consent was not necessary. However, the study was approved by the Human Ethics Committee of the Affiliated Hospital of Jining Medical University.

The study enrolled a total of 1018 participants, with an entry time of December 1, 2013, and a deadline for inclusion of January 1, 2023. Inclusion criteria included the renal tumor was diagnosed as clear cell carcinoma by surgery or puncture pathology. Exclusion criteria for this study included patients who had a history of taking diuretics or hypolipidemic medications, patients who had received treatment with antigout medications.

### 2.3. Variables

In this study, SUA was obtained at baseline and recorded as a continuous variable, using a biochemical automatic analyzer (Cobas c702, Roche; Shanghai, China). The final outcome variable, triglycerides, was obtained using the same process of using a biochemical automatic analyzer (Cobas c702, Roche; Shanghai, China) in accordance with published guidelines and prior research.

The covariates included in this study can be summarized as follows: demographic data, variables affecting SUA or TGs according to previous literature, and variables based on clinical experience. The fully-adjusted model included the following continuous variables obtained at baseline: age, high-density lipoprotein cholesterol (HDL-C), low-density lipoprotein cholesterol (LDL-C), total cholesterol (TC), blood urea nitrogen (BUN), Creatinine (Cr), and body mass index (BMI); as well as categorical variables obtained at baseline: sex, smoking status, hypertension, diabetes, and coronary disease.

### 2.4. Statistical analysis

Continuous variables in our study were presented in 2 forms: mean ± standard deviation for those with normal distribution, and median (min, max) for those with skewed distribution. Categorical variables were expressed in frequency or percentage. To test for differences among the different SUA groups (quartiles), we used the χ^2^ test for categorical variables, 1-way ANOVA test for normally distributed continuous variables, and the Mann–Whitney *U* test for skewed distributed continuous variables. The data analysis process can be summarized in 3 steps. In step 1, we employed univariate and multivariate linear regression models. Three models were constructed: model 1, with no covariates adjusted; model 2, adjusted only for sociodemographic data; model 3, adjusted for model 2 plus other covariates presented in Table 1. In step 2, the penalty spline method was used to construct a fully-adjusted model with a smooth curve fit, exploring the potential linear relationship between SUA and TGs. In step 3, we conducted a sensitivity analysis to ensure the robustness of the data analysis. We converted SUA into a categorical variable and calculated the *P* value for trend. The aim was to verify the results of SUA as a continuous variable and observe the possibility of linearity. The statistical software packages R (http://www.R-project.org, The R Foundation) and EmpowerStats (http://www.empowerstats.com, X and Y Solutions, Inc, Boston, MA) were used for all analyses. *P* values <.05 (2-sided) were considered statistically significant.

**Table 1:** Baseline characteristics of the participants.

SUA (100μmol/L, min - max)	Q1 (0.40–2.45)	Q2(2.46–2.94)	Q3 (2.95–3.53)	Q4(3.54–5.77)	*P* value
Number	244	244	245	247	
Age (yr, mean ± sd)	57.96 ± 10.06	58.19 ± 10.17	57.46 ± 11.34	53.84 ± 11.32	<.001
BMI (kg/m^2^, mean ± SD)	24.74 ± 3.72	24.98 ± 3.27	26.10 ± 3.77	27.01 ± 3.68	<.001
Cr (μmol/L, mean ± SD)	60.85 ± 27.13	68.47 ± 17.09	73.28 ± 32.56	76.31 ± 19.24	<.001
BUN (mmol/L, mean ± SD)	4.80 ± 1.46	5.30 ± 1.34	5.62 ± 1.71	5.68 ± 1.58	<.001
TC (mmol/L, mean ± SD)	1.27 ± 0.98	1.35 ± 0.83	1.50 ± 0.97	1.71 ± 1.03	<.001
HDL-C (mmol/L, mean ± SD)	1.27 ± 0.34	1.26 ± 0.30	1.21 ± 0.28	1.19 ± 0.28	.008
LDL-C (mmol/L, mean ± SD)	2.69 ± 0.69	2.71 ± 0.78	2.83 ± 0.76	2.91 ± 0.83	.004
TGs (mmol/L, mean ± SD)	4.51 ± 0.95	4.53 ± 0.97	4.66 ± 0.92	4.72 ± 1.04	.040
SEX, n (%)					<.001
Male	110 (44.7)	157 (64.3)	179 (73.1)	207 (83.8)	
Female	134 (55.3)	87 (35.7)	66 (26.9)	40 (16.2)	
Smoking status, n (%)					<.001
No	184 (75.4)	163 (66.8)	144 (58.8)	148 (59.9)	
Yes	60 (24.6)	81 (33.2)	101 (41.2)	99 (40.1)	
Alcohol consumption, n (%)					<.001
No	203 (83.2)	185 (75.8)	158 (64.5)	151 (61.1)	
Yes	41 (16.8)	59 (24.2)	87 (35.5)	96 (38.9)	
Hypertension, n (%)					.139
No	165 (67.6)	166 (68.0)	158 (64.5)	146 (59.1)	
Yes	79 (32.4)	78 (32.0)	87 (35.5)	101 (40.9)	
Diabetes, n (%)					<.001
No	194 (79.5)	218 (89.3)	207 (84.5)	228 (92.3)	
Yes	50 (20.5)	26 (10.7)	38 (15.5)	19 (7.7)	
Coronary disease, n (%)					.367
No	215 (88.1)	220 (90.2)	216 (88.2)	228 (92.3)	
Yes	29 (11.9)	24 (9.8)	29 (11.8)	19 (7.7)	

BMI = body mass index, BUN = blood urea nitrogen, Cr = creatinine, HDL-C = high-density lipoprotein cholesterol, LDL-C = low-density lipoprotein cholesterol, SUA = serum uric acid, TC = total cholesterol, TGs =triglycerides.

## 3. Results

### 3.1. Baseline characteristics of selected participants

A total of 1018 participants were included in the data analysis. Table 1 presents the baseline characteristics of the study participants according to the Quartile of SUA. The average age of the participants in this study was 56.92 ± 10.88 years and approximately 68% of the participants were male. There were no statistically significant differences in hypertension and coronary disease among different SUA groups (all *P* values > .05). Participants in the highest SUA group (Q4) had higher values of BMI, Cr, BUN, TC, LDL-C, and TGs compared to those in the other groups. The opposite pattern was observed for HDL-C and age, where the Q4 group had lower HDL-C levels and were younger compared to the other groups.

### 3.2. Unvariate analysis

Table 2 shows the results of univariate analyses. In univariate linear regression, we found that sex, smoking status, alcohol consumption, hypertension, diabetes, coronary disease, BUN, and LDL-C were not associated with TGs. Univariate analysis also revealed that HDL-C (−0.84, −1.04 to 0.64) and age (−0.01, −0.02 to 0.01) were negatively associated with TGs. In contrast, BMI (0.05, 0.04–0.07), Cr (0.00, 0.00–0.01), and TC (0.28, 0.22–0.34) were positively correlated with TGs.

**Table 2:** Univariate analysis for TGs (mmol/L).

Covariate	Statistics	β (95% CI)	*P* value
Sex			
Male	680 (66.80%)	Reference	
Female	338 (33.20%)	−0.10 (−0.23, 0.03)	.1266
Age, yr	56.92 ± 10.88	−0.01 (−0.02, −0.01)	<.0001
BMI, kg/m^2^	25.66 ± 3.70	0.05 (0.04, 0.07)	<.0001
Smoking status			
No	659 (64.73%)	Reference	
Yes	359 (35.27%)	0.07 (−0.06, 0.20)	.2823
Alcohol consumption			
0	722 (70.92%)	Reference	
1	296 (29.08%)	0.07 (−0.06, 0.21)	.2910
Hypertension			
0	658 (64.64%)	Reference	
1	360 (35.36%)	0.11 (−0.02, 0.24)	.0957
Diabetes			
0	881 (86.54%)	Reference	
1	137 (13.46%)	0.10 (−0.08, 0.29)	.2674
Coronary disease			
0	912 (89.59%)	Reference	
1	106 (10.41%)	0.13 (−0.07, 0.34)	.2019
Cr (μmol/L)	69.97 ± 25.44	0.00 (0.00, 0.01)	.0028
BUN (mmol/L)	5.33 ± 1.57	0.03 (−0.01, 0.06)	.1972
TC (mmol/L)	4.61 ± 0.97	0.28 (0.22, 0.34)	<.0001
HDL-C (mmol/L)	1.23 ± 0.30	−0.84 (−1.04, −0.64)	<.0001
LDL-C (mmol/L)	3.05 ± 8.16	0.01 (−0.00, 0.01)	.1011

BMI = body mass index, BUN = blood urea nitrogen, CI = confidence interval, Cr = creatinine, HDL-C = high-density lipoprotein cholesterol, LDL-C = low-density lipoprotein cholesterol, TC = total cholesterol, TGs = triglycerides.

### 3.3. Results of unadjusted and adjusted linear regression

In this study, we employed multivariate linear regression to analyze the independent effects of SUA on TGs. Table 3 presents the effect sizes (β) and 95% confidence intervals for the 3 models that were constructed. In the unadjusted model (model 1), the effect size of SUA on TGs can be explained as the difference in 100 umol/L of SUA associated with mmol/L of TGs. In the fully-adjusted model (Adjust II) (adjusted for all covariates presented in Table 1), for each additional 100 umol/L of SUA, TGs (mmol/L) increased by 0.13 mmol/L (β 0.13, 95% CI 0.07–0.18). In the sensitivity analysis, we converted the continuous variable of SUA to a categorical variable, specifically Quartile of SUA. The *P* value for the trend of SUA with categorical variables in the fully-adjusted model was consistent with the findings when SUA was considered a continuous variable.

**Table 3:** Relationship between SUA (100μmol/L) and TGs (mmol/L) in diferent models.

Variable	Non-adjusted		Adjust I		Adjust II	
	β (95% CI)	*P* value	β (95% CI)	*P* value	β (95% CI)	*P* value
SUA, 100μmol/L	0.24 (0.17, 0.31)	<.0001	0.23 (0.15, 0.31)	<.0001	0.13 (0.07, 0.18)	<.0001
SUA (quartile)						
Q1	Reference		Reference		Reference	
Q2	0.08 (−0.09, 0.25)	.3501	0.09 (−0.08, 0.26)	.3051	0.09 (−0.03, 0.21)	.1419
Q3	0.23 (0.06, 0.40)	.0087	0.23 (0.06, 0.41)	.0088	0.08 (−0.04, 0.20)	.2037
Q4	0.44 (0.27, 0.61)	<.0001	0.42 (0.24, 0.60)	<.0001	0.22 (0.09, 0.35)	.0010

Adjust I adjust for: sex, age. Adjust II adjust for: sex, age, HDL-C, LDL-C, TC, BUN, Cr, BMI, smoking status, hypertension, diabetes, coronary disease.

CI = confidence interval, SUA = serum uric acid, TGs = triglycerides.

### 3.4. The results of the association between SUA and TGs.

**Figure 1.**
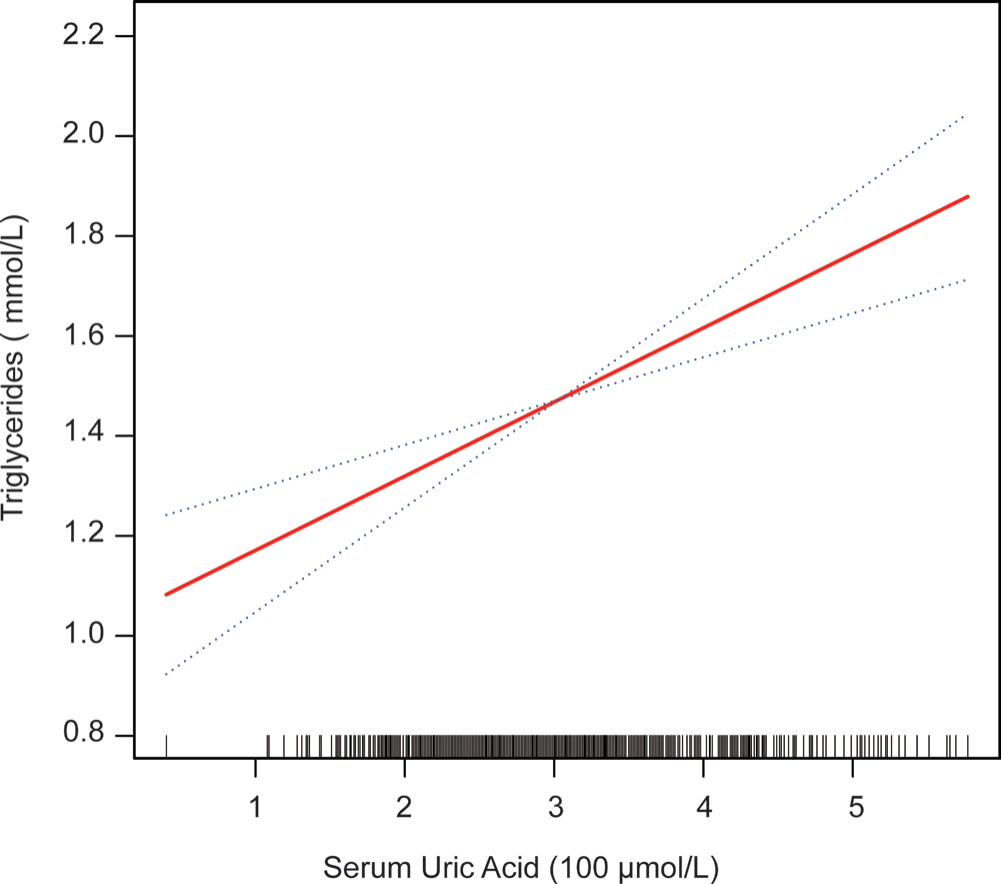
Association between SUA (100 umol/L) and TGs (mmol/L). The solid red line represents the smooth curve fit between the variables. Blue bands represent the 95% confidence interval of the fit. The model was adjusted for age, HDL-C, LDL-C, TC, BUN, Cr, BMI, sex, smoking status, hypertension, and coronary disease. BMI = body mass index, BUN = blood urea nitrogen, Cr = creatinine, HDL-C = high-density lipoprotein cholesterol, LDL-C = low-density lipoprotein cholesterol, SUA = serum uric acid, TC = total cholesterol, TGs = triglycerides.

In this study, we also analyzed the relationship between SUA and TGs using a smooth curve and a generalized additive model (Fig. 1). After adjusting for age, HDL-C, LDL-C, TC, BUN, Cr, BMI, sex, smoking status, hypertension, and coronary disease, the results showed that the relationship between SUA and TGs was linear.

## 4. Discussion

Our study is one of the largest cross-sectional studies that explored the association between SUA and TGs in a Chinese population with ccRCC. We found a positive association between SUA and TGs after adjusting for other covariates. Our results showed that there was a significant increase in TGs by 0.23 mmol/L for each additional unit of 100 umol/L of SUA. As more individuals in the population have metabolic abnormalities, this may aggravate the bias in the detection of TGs. Therefore, to confirm the robustness of the results, we included SUA, the most relevant variable, to construct smooth curve models.

Chu Y et al^[24]^ discovered a nonlinear association between SUA and TG levels in a sample of 1095 short children and adolescents. Similar findings were also reported by Ma W.^[21]^ Also, A higher level of SUA, may be associated with a higher TG in patients with type 2 diabetes mellitus.^[25]^ The consistent conclusions drawn from our study. This implies that SUA might have an essential role in the human body beyond its known function as a waste product of purine metabolism. Moreover, SUA’s multifaceted physiological effects, such as antioxidant effects and mediation of autoimmune diseases, are indicative of the crucial role it could play in the human body.^[26,27]^ However, the study by Ghamri RA et al^[23]^reported no significant association between SUA concentration and lipid profile parameters. We reviewed these studies and speculated that the differences in results may be due to the following factors: Differences in the study population. The studies inconsistent with our findings focused on the city of Jeddah; These studies did not examine the potential nonlinear relationship between SUA and lipid profile parameters; These studies did not take into account the impact of confounding variables such as Cr, BUN, smoking status, alcohol consumption, hypertension, diabetes, and coronary disease on the SUA and TGs relationships when adjusting covariates.

The mechanism underlying the observed association between elevated SUA levels and elevated lipid levels is thought to be related to the promotion of lipid peroxidation, generation of oxygen free radicals, and inflammation of the blood vessel walls caused by high levels of SUA. Additionally, excessive concentrations of SUA are generally considered to be a mediator of inflammatory endocrine disorders in adipose tissue, which may be a significant contributing factor to the development of dyslipidemia.^[28]^ Previous studies have demonstrated that elevated levels of SUA promote the formation of foam cells, decrease cell viability, and increase iron accumulation and lipid peroxidation in macrophages.^[29]^ Furthermore, Experimental research has suggested that uric acid may induce hepatic fat accumulation through the activation of the ROS/JNK/AP-1 pathway.^[30]^

Some strengths of our study include the following: We had a relatively large sample size compared with previous studies on the topic; We addressed the potential linear relationship between SUA and TGs and further investigated this relationship; Our study may be subject to potential confounding, given its observational nature, but we employed strict statistical adjustment methods to minimize residual confounding; We treated the target independent variable as both a continuous variable and a categorical variable, which reduced contingency in the data analysis and enhanced the robustness of the results.

There are some limitation in the present study include: Our research subjects consisted solely of individuals with ccRCC, which might limit the generalizability and extrapolation of our findings to other populations; Because we exclude patients who were already taking diuretics or hypolipidemic medications, and patients using antigout medications. As a result, our findings may not be applicable to these populations.

This study holds valuable clinical significance as it is the first of its kind to observe the independent association between SUA and TGs in ccRCC. The implications of the findings may help guide future research in developing diagnostic or predictive models related to TGs.

## Acknowledgments

The authors thank all the staff members in our institution

## Author contributions

**Formal analysis:** Dong Yue.

**Investigation:** Dong Yue.

**Methodology:** Jun Tian.

**Resources:** Dong Yue, Jun Tian.

**Validation:** Dong Yue.

**Writing – original draft:** Dong Yue.

**Writing – review & editing:** Jun Tian.
